# Epidemiological data of falciparum malaria in Ado-Odo/Ota, Southwest Ogun State, Nigeria

**DOI:** 10.1016/j.dib.2018.06.002

**Published:** 2018-06-09

**Authors:** I.Ruth Diji-geske, I.Grace Olasehinde, Irawo Fadinad, Damola Arogundade, Precious Darby

**Affiliations:** Department of Biological Sciences, Covenant University, Ota, Nigeria

**Keywords:** PCR, Polymerase chain reaction, RFLP, Restriction fragment length polymorphism, Malaria, Prevalence, *Plasmodium falciparum*, Resistance genes

## Abstract

In this data article, Blood and corresponding saliva samples from subjects presenting with fever and parasetaemia ≥2000 were obtained from selected hospitals in Ado-Odo/Ota, Ogun State over a period of two years and analyzed using Polymerase chain reaction-Restriction fragment Length Polymorphism (PCR/Nested PCR-RFLP) to detect genetic mutations of *Plasmodium falciparum* chloroquine resistance transporter *(Pfcrt), Plasmodium falciparum* multidrugs resistance (*Pfmdr1) and non-synonymous* Pkelch (pk13) mutated genes. The study confirmed the presence of resistance genes in the blood and saliva samples collected from the study site.

**Specification Table**

**Table:** 

Subject area	Microbiology
More specific subject area	Epidemiology of malaria
Type of data	Tables and graph
How data was acquired	Sample collection, Microscopy, PCR analysis
Data format	Raw, analyzed
Experimental factors	DNA extraction from blood and saliva samples of subjects presenting with fever and parasitaemia of ≥2000 parasites/ul of blood in selected health facilities.
Experimental features	PCR was used to detect of *Plasmodium falciparum* parasites and resistance genes.
Data source location	Medical diagnostics laboratory Covenant University Medical center and Molecular Research laboratory, Covenant University, Ota, Nigeria.
Data accessibility	Within this research
Related research article	Olasehinde GI, Ojurongbe OO, Fagade EO, Ruchi S, Egwari LO, Ajayi AA, Adeyeba OA. Detection of Molecular Markers of Antimalarial Drug Resistance in Plasmodium Falciparum from South-Western Nigeria*. Covenant Journal of Physical and Life Sciences;* 2014 1(2): 61- 75.

**Value of the data**

• The data provides an epidemiology on falciparum malaria and resistance genes in the study site.
• The data set provides researchers with a platform for enhanced studies in the production of non-invasive malaria test kit using saliva.
• The data establishes the need for improvement of existing drugs/ development of new ones.

## 1 Data

This data article presented and analyzed incidence and prevalence of *Plasmodium falciparum* and resistance genes (*Pfcrt, Pfmdr1 and Pk*13) in ADO-Odo/Ota, Ogun State. It also examined the prospective of saliva to serve as a non-invasive diagnostic method for malaria diagnosis [1]. This data will encourage pragmatic monitoring and surveillance of falciparum malaria in the research area as recommended by the WHO׳s recommendation [2]. It also provides researchers with a platform for enhanced studies in the production of non-invasive malaria test kit using saliva.

## 2 Experimental design, materials and methods

**Fig. 1:**
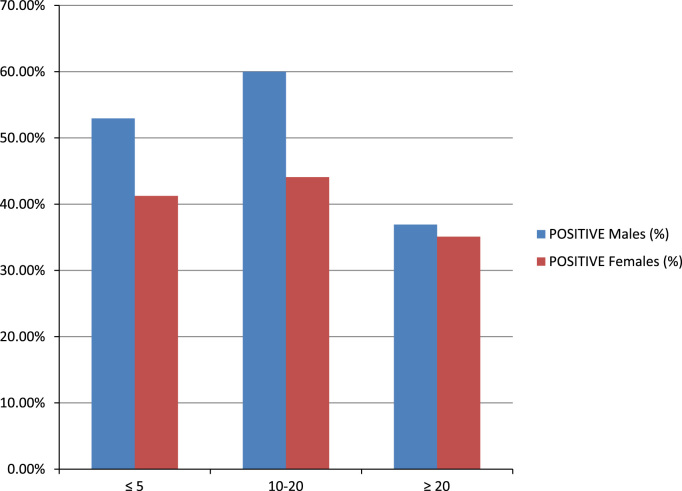
Incidence of *P. falciparum* malaria infection in males against females.

**Fig. 2:**
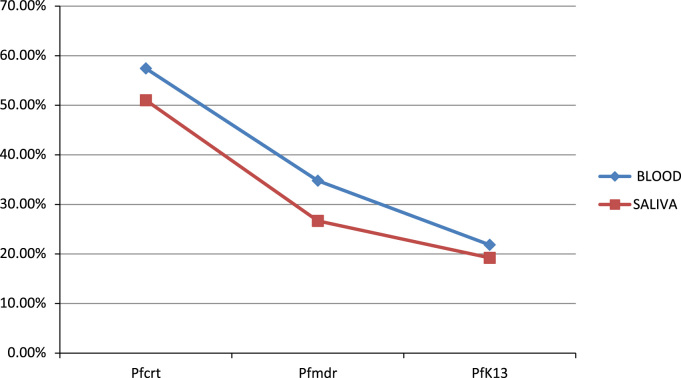
Resistance genes detected in blood and saliva samples.

**Fig. 3:**
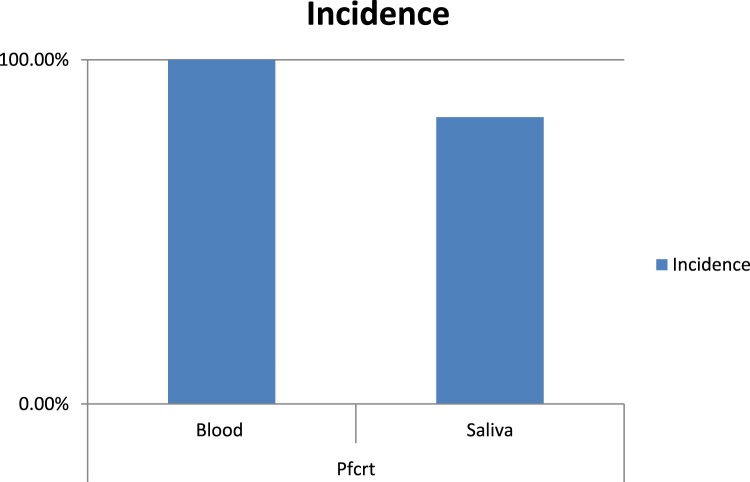
*Pf*crt gene in blood and corresponding saliva samples.

Samples of blood and corresponding saliva from subjects were collected from various hospitals in Ado-Odo Local government area of Ogun State for three years. The study group for the research cut across sexes of different age groups of patients presenting with fever and parasitaemia of ≥2000 parasites/ul of blood. Ethical approval for this study was obtained from the Covenant University Biological Sciences Ethical Review Committee (CUBIOSCREC). Informed consent was obtained from all participants. Where participant was a minor, consent was obtained from participant׳s guardian. Parasite identification and infective stage was determined using microscopy study. For molecular studies, Parasite DNA from blood and saliva was extracted using a genomic DNA extraction kit. Nested PCR-RFLP using the specific primers - *Pfcrt*, *Pfmdr1* and Pk13 gene was carried out [3], [4], [5]. Amplicons were sequenced directly by using each primer for target gene amplification. Data were analysed and presented as follows; Table 1 shows the incidence of *P. falciparum* infection in Ado-Odo/Ota Local government area of Ogun State, Nigeria in the year 2015. Table 2 shows the incidence of falciparum malaria in the year 2016. Table 3 shows the incidence of falciparum malaria in the year 2017. Table 4 shows the prevalence of falciparum malaria within two years. Table 5 shows the prevalence of resistance genes. Fig. 1 presents the incidence of *P. falciparum* malaria infection in males and females. Fig. 2 presents the resistance genes detected in blood and saliva samples. Fig. 3 presents *Pf*crt gene detected in blood and exact number of corresponding saliva samples.

**Table 1:** Incidence of falciparum malaria in Ado-Odo/Ota, Ogun State (2015).

Number of samples collected				Number of positive cases			
AGE	Male	Female	Total	Male	Female	Total	% Incidence
≤5	43	56	99	24	20	44	44.44
10–20	233	276	509	144	114	258	50.69
≥20	126	132	258	47	33	80	31.01
Total	402	464	866	215	167	382	44.11

**Table 2:** Incidence of falciparum malaria in Ado-Odo/Ota, Ogun State (2016).

Number of samples collected				Number of positive cases			
Age	Male	Female	Total	Male	Female	Total	% Incidence
≤5	8	15	23	5	8	13	56.52
10–20	39	35	74	31	21	52	70.27
≥20	38	30	68	14	23	37	54.41
Total	85	80	165	50	52	102	61.82

**Table 3:** Incidence of falciparum malaria in Ado-Odo/Ota, Ogun State (2017).

Number of samples collected				Number of positive cases			
Age	Male	Female	Total	Male	Female	Total	% Incidence
≤5	17	9	26	7	5	12	46.15
10–20	41	47	88	12	18	30	34.09
≥20	27	41	68	8	12	20	29.41
Total	85	97	182	27	35	62	34.07

**Table 4:** Prevalence of falciparum malaria in Ado-Odo/Ota, Ogun State (2015–2017).

Number of samples collected				Number of positive cases			
Age group	Male	Female	Total	Male	Female	Total	% Total
≤5	68	80	148	36	33	69	46.62
10–20	313	358	671	187	153	340	50.67
≥20	191	203	394	69	68	137	34.77
Total	572	641	1213	292	254	546	45.01

**Table 5:** Prevalence of resistance genes in Ado-Odo/Ota, Ogun State.

**GENES**	**No of samples**		**Positive samples**		**Incidence (%)**	
	**Blood**	**Saliva**	**Blood**	**Saliva**	**Blood**	**Saliva**
***Pfcrt***	71	35	34	11	47.89	31.43
***Pfmdr***	46	30	16	8	34.78	26.67
***PfK13***	87	18	19	8	21.84	44.44
***Total***	204	83	69	27	33.82	32.53
